# Mitral valve sub valvular apparatus preservation in pure or predominant rheumatic mitral stenosis

**DOI:** 10.1186/1749-8090-10-S1-A2

**Published:** 2015-12-16

**Authors:** Abdelmalek Bouzid, Salim Chibane, Mohamed Atbi, Halima Larbi, Boukri Hamouda, Redha Djilali-Sayeh, Youcef Larabi, Tarek Hamdi, Sami Bouchenafa, Ramdan A Ould Abderrahmane

**Affiliations:** 1 Department of Cardiac Surgery, EHU 1er Novembre 54, Oran 31000, Algeria

## Background/Introduction

Surgery of the mitral valve is an important part of the surgical activity at Algerian heart surgery departments.

In many cases the lesions exceed the possibility of native valve preservation, thus requiring the replacement thereof.

When replacing, conservation of the subvalvular apparatus or not remains a question which had a real interest, which was reflected in the significant number of studies on the subject.

The majority of these studies have demonstrated the value of subvalvular apparatus preservation on the future of ventricular function in the immediate and distant; however almost all of these studies have concerned patients with mitral insufficiency lesions.

## Aims/Objectives

Our aim is to evaluate the impact of mitral valve subvalvular apparatus preservation on left ventricular function after valve replacement in patient with predominant or pure mitral stenosis

## Method

77 patients underwent a mitral valve replacement for mitral valve stenosis, 54 patients (group1) had no preservation, 23 patients (group 2) had partial sub valvular preservation; there were no significant differences between the two groups in pre-operative parameters (age, sex, functional status, AF, LVEDD, LVESD, LVEF).

The mean follow up was 19 months.

## Results

In all of the postoperative study parameters, no significant difference was found between groups either in terms of mortality, left or right ventricular function, LVEF (62.98 +/- 2.65% vs 61 +/- 4.20 %); LVEDD (47.3 +/- 1.4 mm vs 46.1 +/- 2.6 mm); LVESD (30.8 +/- 1.7 mm vs 30.9 +/- 2.6 mm); TAPSE (15.78 +/- 0.88 mm vs 15.65 +/- 1.0 mm), Sa (10.58 +/- 0.47 cm/s vs 10.35 +/- 0.77 cm/s) respectively for group 1 and group 2.

## Discussion/Conclusion

The short- and medium-term results of our study did not demonstrate the interest of preserving the sub valvular apparatus in mitral valve replacement surgery on pure or predominant rheumatic mitral stenosis; however a longer follow-up is need to evaluate the impact on either left or right ventricular function.

